# Triple herbal extract DA-9805 exerts a neuroprotective effect via amelioration of mitochondrial damage in experimental models of Parkinson’s disease

**DOI:** 10.1038/s41598-018-34240-x

**Published:** 2018-10-29

**Authors:** Jin Seok Jeong, Ying Piao, Sora Kang, Minuk Son, Young Cheol Kang, Xiao Fei Du, Jayoung Ryu, Young Woong Cho, Hai-Hua Jiang, Myung Sook Oh, Seon-Pyo Hong, Young J. Oh, Youngmi Kim Pak

**Affiliations:** 1 R&D Center of Dong-A ST, Yong-in, Kyungki-do, 17073 Korea; 2 Department of Systems Biology, College of Life Science and Biotechnology, Yonsei University, Seoul, 03722 Korea; 3 Department of Neuroscience, Graduate School, Kyung Hee University, Seoul, 02447 Korea; 4 Department of Oriental Pharmaceutical Science, College of Pharmacy, Kyung Hee University, Seoul, 02447 Korea; 5 Department of Physiology, College of Medicine, Kyung Hee University, Seoul, 02447 Korea; 6 Present Address: Department of Emergency, Yanbian University Hospital, Yanji City, Jilin Province 133000 China

## Abstract

Moutan cortex, Angelica Dahurica root, and Bupleurum root are traditional herbal medicines used in Asian countries to treat various diseases caused by oxidative stress or inflammation. Parkinson’s disease (PD) has been associated with mitochondrial dysfunction, but no effective treatment for mitochondrial dysfunction has yet been identified. In this study we investigated the neuroprotective effects of the triple herbal extract DA-9805 in experimental models of PD. DA-9805 was prepared by extracting three dried plant materials (Moutan cortex, Angelica Dahurica root, and Bupleurum root in a 1:1:1 mixture) with 90% ethanol on a stirring plate for 24 h at room temperature and fingerprinted using high-performance liquid chromatography. 1-methyl-4-phenyl-1,2,3,6-tetrahydropyridine (MPTP) and its active metabolite 1-methyl-4-phenylpyridinium (MPP^+^), which both exert neurotoxic effects on dopaminergic neurons by inhibiting mitochondrial oxidative phosphorylation (OXPHOS) complex I, were used to make experimental models of PD. In MPP^+^-treated SH-SY5Y cells, DA-9805 ameliorated the suppression of tyrosine hydroxylase expression and mitochondrial damage on OXPHOS complex 1 activity, mitochondrial membrane potential, reactive oxygen species (ROS) generation, and oxygen consumption rate. In the MPTP-induced subacute PD model mice, oral administration of DA-9805 recovered dopamine content as well as bradykinesia, as determined by the rotarod test. DA-9805 protected against neuronal damage in the substantia nigra pars compacta (SNpc) and striatum. In both *in vitro* and *in vivo* models of PD, DA-9805 normalized the phosphorylation of AKT at S473 and T308 on the insulin signaling pathway and the expression of mitochondria-related genes. These results demonstrate that the triple herbal extract DA-9805 showed neuroprotective effects via alleviating mitochondria damage in experimental models of PD. We propose that DA-9805 may be a suitable candidate for disease-modifying therapeutics for PD.

## Introduction

Parkinson’s disease (PD) is a chronic neurodegenerative disorder caused by a progressive loss of dopaminergic neurons projecting from the substantia nigra pars compacta (SNpc) to the striatum, which leads to decreased dopamine levels in the basal ganglia^1^. This decrease in dopamine levels is associated with several adverse clinical motor symptoms, including bradykinesia, resting tremor, rigidity, and postural instability. In addition, the formation of Lewy bodies, which consist of abnormal aggregated α-synuclein, in dopaminergic neurons is regarded as a key pathological hallmark of PD.

Although the etiology of the selective loss of dopaminergic neurons is not understood, several genetic and environmental risk factors that trigger the progression of PD have been identified^2^. It has been proposed that these risk factors converge on mitochondrial dysfunction and subsequently lead to dopaminergic neurodegeneration. More specifically, many of the causative genes associated with familial PD were verified to be specific to mitochondrial function^3^. For example, several genes [e.g., α-synuclein, parkin, PTEN-induced putative kinase 1 (PINK1), DJ-1, and leucine-rich repeat kinase 2 (LRRK2)] associated with PD are involved in the generation of reactive oxygen species (ROS) and proteolysis in the mitochondrial outer membrane^4^. Another PD-associated gene, PARK13 (HTRA2/OMI), is localized within the mitochondrial inner membrane^4^. PINKI, parkin, and DJ-1 are involved in maintaining both mitochondrial dynamics and the mitochondrial network^5^.

Studies from sporadic cases of PD also support the hypothesis that mitochondrial dysfunction comprises a major cause of dopaminergic neurodegeneration^6^. The neurotoxins MPTP (1-methyl-4-phenyl-1,2,3,6-tetrahydropyridine) and its active metabolite MPP^+^ (1-methyl-4-phenyl-2,3-dihydropyridinium ion) have been widely used to establish experimental models of PD due to their selective inhibition of mitochondria electron transport system complex 1. Exposure to insecticide paraquat and herbicide rotenone, which are also known to suppress the mitochondrial electron transport system, is linked to PD in animal^7^ and human studies^8,9^. This evidence supports the idea that the selective death of dopaminergic neurons due to mitochondrial dysfunction may be a cause of PD; hence, recovery of mitochondrial activity may be an important target for the treatment of PD^10^.

Moutan cortex (MC) is the root bark of *Paeonia suffruticosa Andrews*, which is an herbal medicine containing paeonol, paeoniflorin, oxypaeoniflorin, garlic acid and paeoniflorigenone. MC is traditionally used in Asian countries for relaxation, analgesia, and the treatment of inflammatory disease. In addition, MC has been shown to have antioxidant^11^, anti-cancer^12^ and hypoglycemic^13^ effects. Angelica Dahurica Root (ADR) is the dried root of *Angelica dahurica Bentham et Hooker f*. (or *Angelica dahurica Bentham et Hooker f. var. formosana Shan et Yuan*), which is native to Korea, China, and Japan. The major bioactive compounds of ADR include imperatorin, isoimperatorin, oxypeucedanin, phellopterin, and byakangelicol. ADR has been used to treat sweating, pain, cold, headache, and toothache^14,15^ and also has a sedative effect. Bupleurum Root (BR) is the root of *Bupleurum falcatum Linne* or its variants (Umbelliferae). The major bioactive compounds of BR include saikosaponin A, C, D; triterpenoids; flavonoids; polyacetylenes; and polysaccharides. BR has been shown to have neuroprotective^16^, anti-cancer^17^, and anti-inflammatory^18,19^ effects.

Our previous work demonstrated that ethanol extract of MC, ADR, or BR has significant neuroprotective effects in various models of neurodegeneration: MC extract restores MPP^+^-induced mitochondrial damage in rat primary dopaminergic neurons^20^, whereas the extract of BR blocked lipopolysaccharide (LPS)-induced chemokine/cytokine productions in microglial cells^18^ and the extract of ADR recovered thapsigargin- or tunicamycin-induced endoplasmic reticulum (ER)-stress in SH-SY5Y cells (unpublished data). The combined water extract of MC, ADR and BR showed protective effects on dopaminergic neurons by regulating Nurr1 in the mouse model of PD^21^. But its effect on mitochondrial activity of dopaminergic neuron has not been studied. Based on these findings, we prepared a 90% ethanol extract named DA-9805, which is suitable for pharmaceutical manufacturing, in combination with MC with BR and ADR. The present study examined whether DA-9805 has neuroprotective effects on experimental PD models of MPTP-injected mice and MPP^+^-treated neuronal cells compared to reference PD drugs. Our biochemical and behavioral findings indicate that DA-9805 protects dopaminergic neuronal cells from neurotoxicity via a protective effect against mitochondrial dysfunction better than reference drugs.

## Results

### Standardization and UHPLC fingerprinting of DA-9805

**Figure 1:**
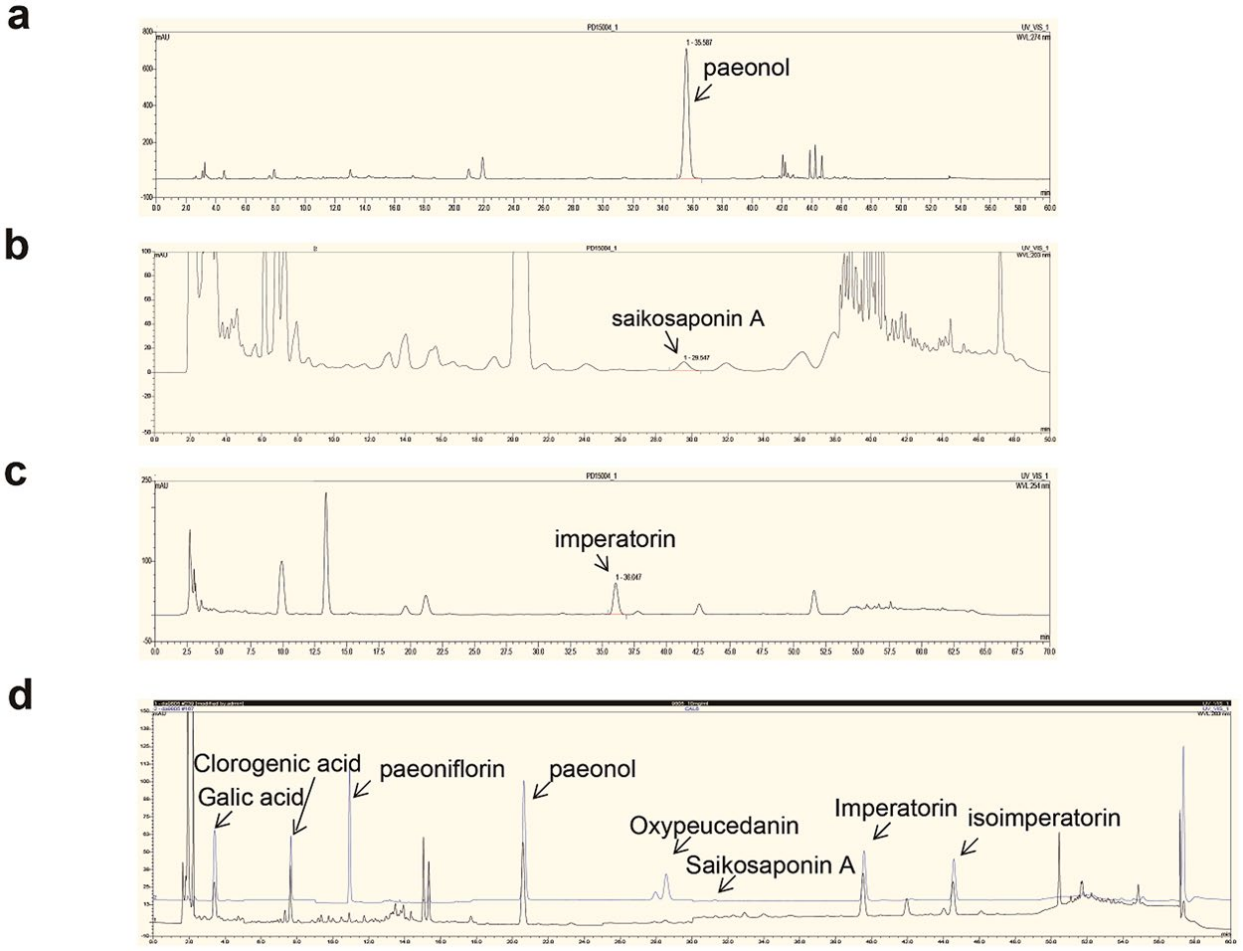
UHPLC chromatograms. DA-9805 was analyzed by ultra-high performance liquid chromatography (UHPLC) for (**a**) paeonol, (**b**) saikosaponin A, and (**c**) imperatorin. (**d**) Comparative chemical fingerprinting of DA-9805 (black) with standard mixtures of eight ingredients (blue): gallic acid, chlorogenic acid, paeoniflorin, paeonol, oxypeucedanin, saikosaponin A, imperatorin, and isoimperatorin.

DA-9805 batch MB1601 was standardized by UHPLC analysis to specify the contents of the index constituents (i.e., paeonol for MR, saikosaponin A for BR, and imperatorin for ADR) in DA-9805 (Fig. 1a,c). Concentration ranges of paeonol, saikosaponin A and imperatorin in DA-9805 were 2.026–6.079%, 0.196–0.588% and 0.299–0.897%, respectively. The UHPLC profiles of the eight ingredients (gallic acid, chlorogenic acid, paeoniflorin, paeonol, oxypeucedanin, saikosaponin A, imperatorin, and isoimperatorin) are presented with that of DA-9805 in Fig. 1d. The constituents of DA-9805 were well separated under the established UHPLC conditions. The eight ingredients were identified by comparing the retention times and UV spectrum with standard mixtures. To obtain a stable and reproducible chromatographic fingerprint of the medicinal extract for quality control purposes, the UHPLC fingerprint analysis was validated using the retention times and peak areas of the eight ingredients.

### DA-9805 protected MPP+-induced cellular and mitochondrial damage in SH-SY5Y cells

**Figure 2:**
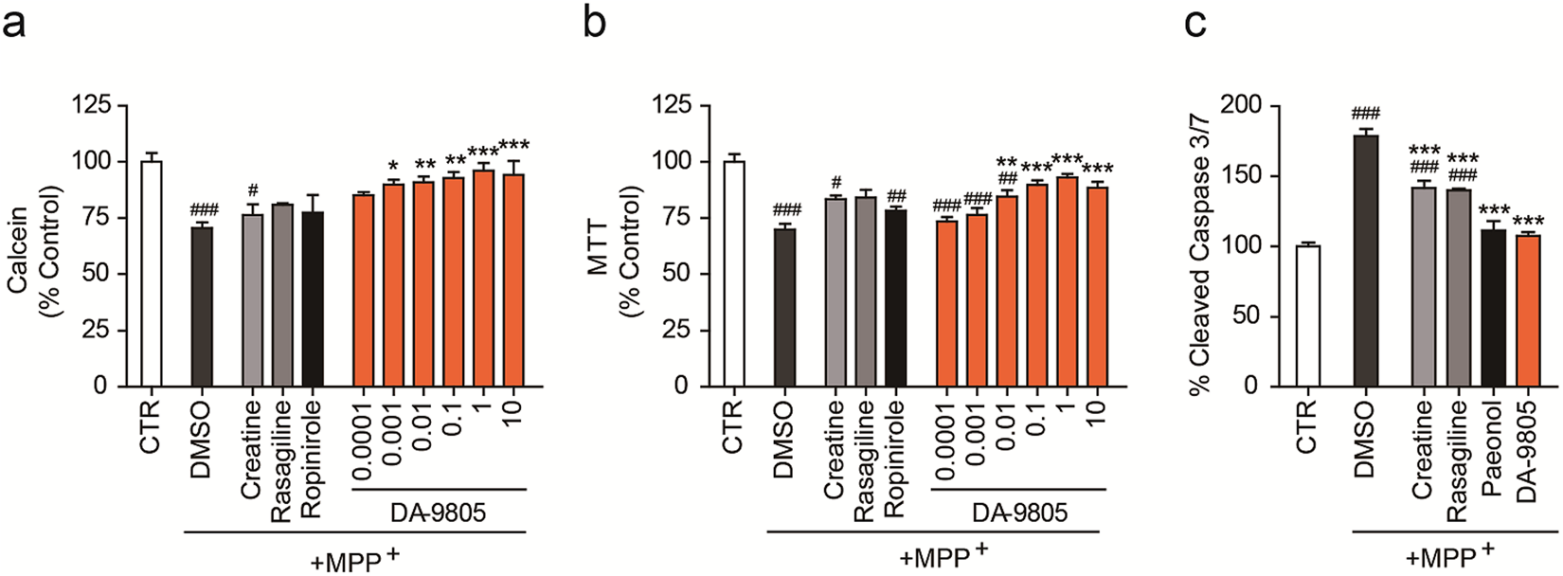
Recovery of cell viability by DA-9805. SH-SY5Y cells were placed in a 96-well plate and pre-treated with various concentrations of DA-9805 (μg/ml) or reference drugs (50 µM creatine, 100 nM rasagiline, or 50 µM ropinirole) for 4 h. The cells were incubated with 1 mM MPP^+^ for 20 h, and then cell viabilities were analyzed. (**a**) Calcein cell permeability assay. (**b**) MTT NADH dehydrogenase (complex 1) activity. (**c**) Caspase 3/7 activity. DA-9805 (1 μg/ml) was used. All values are reported as a percentage of the control (CTR). The data are plotted as the mean ± SEM (n = 6). ^#^*p* < 0.05, ^##^*p* < 0.01, ^###^*p* < 0.001 vs. CTR; *p < 0.05, **p < 0.01, and ***p < 0.001 vs. MPP^+^-treated DMSO. *p* values are from a one-way ANOVA followed by Tukey’s test.

**Figure 3:**
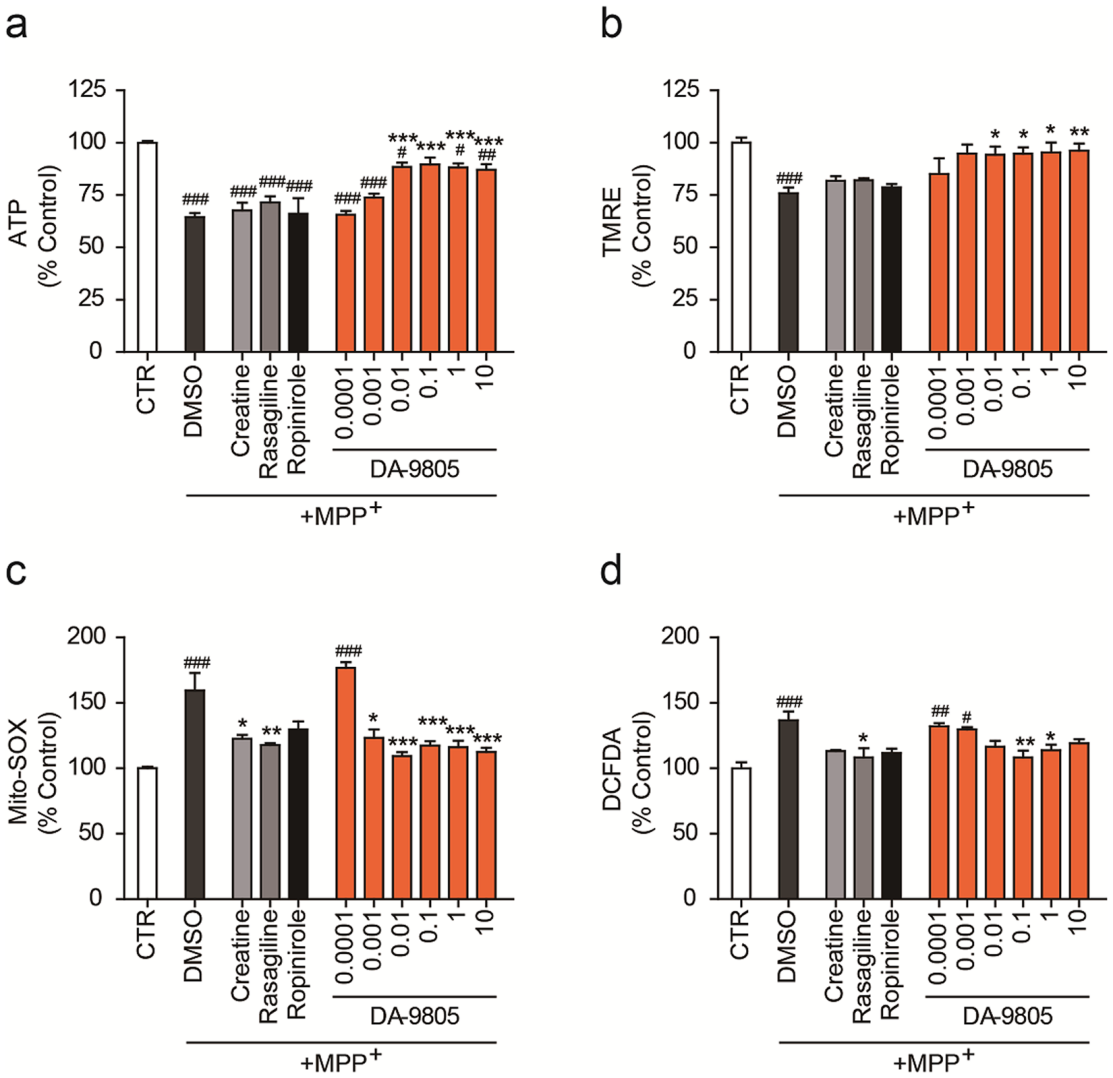
DA-9805 restores mitochondrial activity. SH-SY5Y cells were placed in a 96-well plate and treated with DA-9805 (μg/ml) or reference drugs (50 µM creatine, 100 nM rasagiline, or 50 µM ropinirole) for 4 h. The cells were incubated with 1 mM MPP^+^ for 20 h, and the mitochondrial activity was analyzed. (**a**) Intracellular ATP content. (**b**) TMRE-mediated mitochondrial membrane potential. (**c**) MitoSox-based mitochondrial superoxide generation. (**d**) DCF-DA-based total reactive oxygen species (ROS) generation. All values are reported as a percentage of the control (CTL). The data are plotted as the mean ± SEM (n = 6). ^#^*p* < 0.05, ^##^*p* < 0.01, ^###^*p* < 0.001 vs. CTR; *p < 0.05, **p < 0.01, and ***p < 0.001 vs. MPP^+^-treated DMSO. *p* values are from a one-way ANOVA followed by Tukey’s test.

Dose-dependent effects of DA-9805 on MPP^+^-induced cellular and mitochondrial damage in SH-SY5Y cells were evaluated using cell-based mitochondrial activity assays. SH-SY5Y cells were pre-treated with either DA-9805 at different concentrations (0.0001~10 µg/mL) or one of three reference compounds (creatine, 50 µM; rasagiline, 100 nM; or ropinirole, 50 µM) prior to MPP^+^ treatment. As shown in Fig. 2, DA-9805 increased cell viability (calcein) and NADH dehydrogenase complex 1 activity (MTT) in a dose-dependent manner and decreased caspase3/7-mediated apoptosis. From the concentration of 0.01 µg/mL, DA-9805 protected cell viability better than any of the reference compounds. Similarly, DA-9805 increased the mitochondrial activity of intracellular ATP content and TMRE-mediated mitochondrial membrane potential in MPP^+^-treated SH-SY5Y cells (Fig. 3). ROS production by mitochondria (MitoSox) and cell (DCF-DA) also were reduced toward control levels by DA-9805. In most cases, DA-9805 was more protective against the damage induced by MPP^+^ compared to the reference compounds.

The protective effects of DA-9805 on mitochondrial activity were confirmed in primary cortical neuronal cells, which were isolated from the rat embryonic brain at E18 (Supplementary Fig. S1). Both DA-9805 (1 μg/ml) and paeonol (1 μg/ml), one of the active components of MC, were protective against MPP^+^-mediated cellular and mitochondrial damage. These findings suggest that DA-9805 containing 2~6% paeonol content is more effective than paeonol in protecting against MPP^+^-induced mitochondrial dysfunction in primary cortical neuronal cells.

### DA-9805 normalizes MPP^+^-induced oxygen consumption rate reduction in SH-SY5Y cells

**Figure 4:**
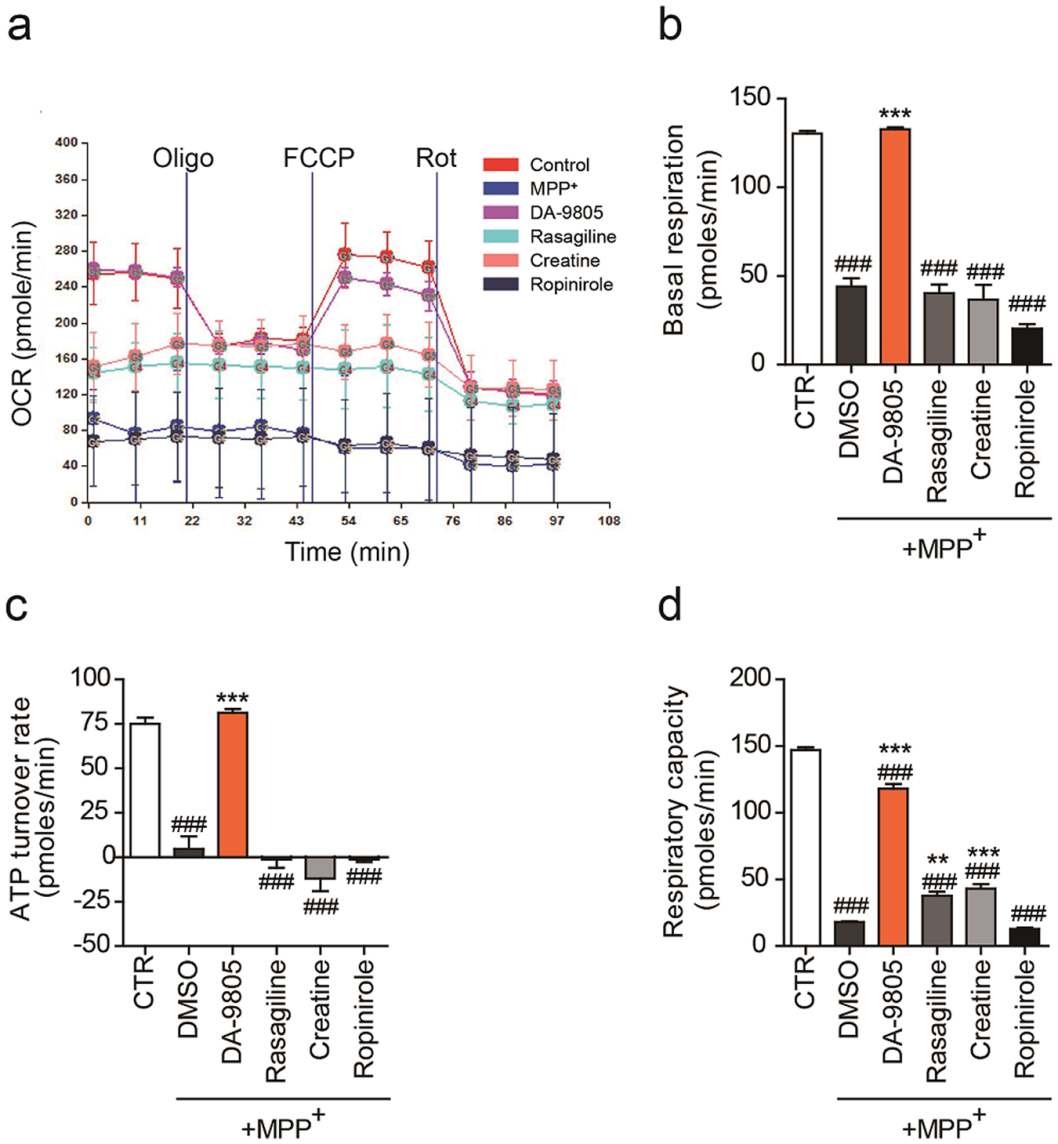
DA-9805 restores mitochondrial oxygen consumption rate. SH-SY5Y cells were placed in an XF-24 microplate and treated with DA-9805 (1 μg/ml) or reference drugs (50 µM creatine, 100 nM rasagiline, or 50 µM ropinirole) for 4 h. The cells were incubated with 1 mM MPP^+^ for 20 h, and oxygen consumption rates (OCR) were analyzed using a Seahorse XF-24 analyzer. Oligomycin (Oligo), FCCP, and rotenone (Rot) were consecutively injected to obtain mitochondrial respiration capacities. (**a**) OCR profiles. (**b**) Basal respiration OCR. (**c**) ATP turnover rate (basal OCR - oligomycin-inhibited OCR). (**d**) Total respiratory capacity (FCCP-induced OCR). The data are plotted as the mean ± SEM (n = 3). One way ANOVA with Tukey post-hoc testing analysis was performed. ^#^*p* < 0.05, ^##^*p* < 0.01 vs. CTR; **p < 0.01, and ***p < 0.001 vs. MPP^+^-treated DMSO. *p* values are from a one-way ANOVA followed by Tukey’s test.

OCR is a direct measurement of cellular mitochondrial function under physiologic conditions. Treatment with oligomycin, an ATP-synthase inhibitor, reveals the amount of oxygen consumption needed for ATP synthesis (oligomycin-mediated ATP turnover rate). FCCP, an uncoupling agent, induces membrane potential dissipation and rapid oxygen consumption to maintain cellular energy balance. FCCP-induced respiratory capacity is defined as the maximal respiratory capacity of the mitochondria. Rotenone, a mitochondrial complex I inhibitor, blocked mitochondrial respiration completely, demonstrating non-mitochondrial respiration. Thus, OCR profiles in Fig. 4a showed that MPP^+^ treatment disrupted the OCR of SH-SY5Y cells. Only cells pre-treated with DA-9805 prior to administration of 1 mM MPP^+^ exhibited normal cellular respiration, including basal OCR (Fig. 4b), ATP turnover rate (Fig. 4c), and respiratory capacity (Fig. 4d). Other reference drugs (creatine, rasagiline, or ropinirole) did not change the MPP^+^-induced blockage of OCR (Fig. 4). These findings suggest that pre-treatment with DA-9805 protects neuronal cells from mitochondrial impairment by MPP^+^.

### *In vivo* effects of DA-9805 on motor function and striatal dopamine levels in an MPTP-injected subacute PD model mice

**Figure 5:**
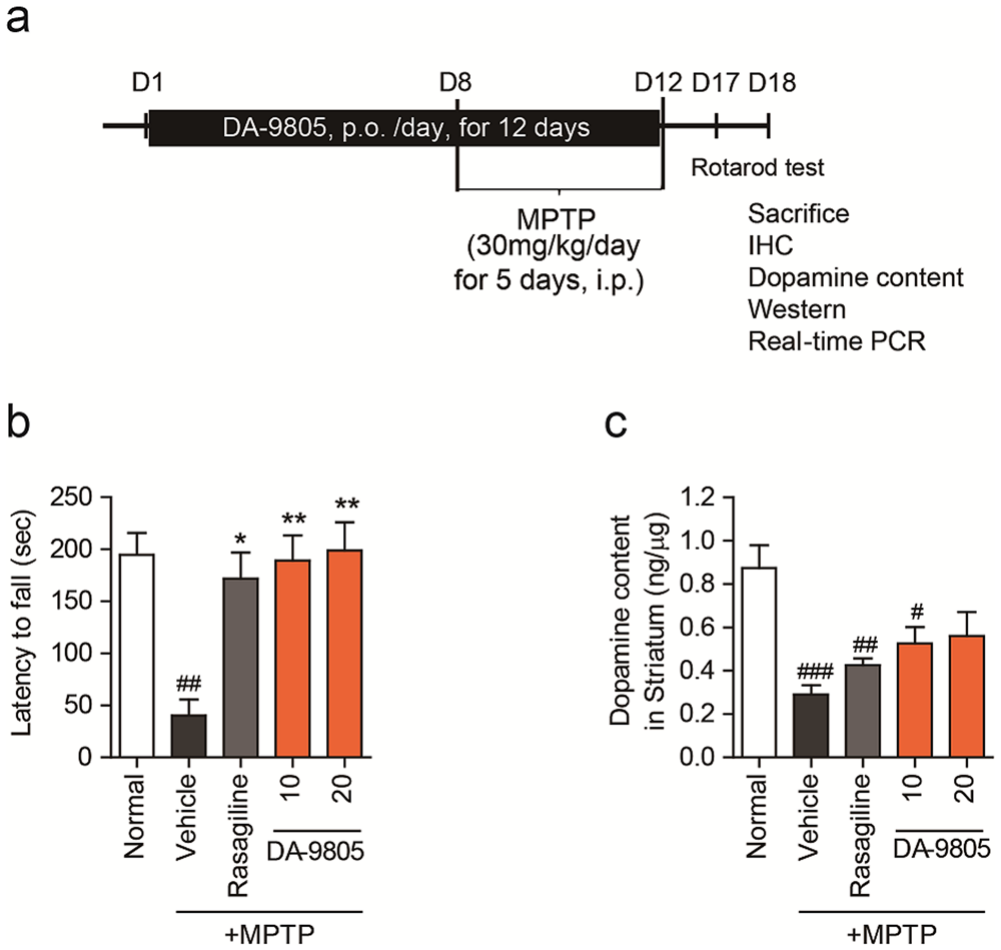
Effects of DA-9805 on behavioral deficits and striatal dopamine levels in an MPTP-induced subacute PD model mice. (**a**) Schema of the experimental design. C57BL/6 mice (male 8–10 weeks old) were orally administered DA-9805 (0.3~20 mg/kg/day), rasagiline (0.05 mg/kg/day), or 3% hydroxypropylmethylcellulose (HPMC) vehicle for 12 consecutive days. All groups except for the normal control group were injected MPTP intraperitoneally at 30 mg/kg/day for five days starting on day 8. On day 17, the rotarod test was performed. On day 18, mice were sacrificed and tissues were prepared for immunohistochemical (IHC) or biochemical detection. (**b**) Motor behavioral deficits were assessed by the rotarod test on day 17. (**c**) Dopamine contents were measured in the striatum on day 18 using high-performance liquid chromatography (HPLC). The data are presented as the mean ± SEM (n = 6). ^#^*p* < 0.05, ^##^*p* < 0.01, ^###^*p* < 0.001 vs. Normal; *p < 0.05, **p < 0.01, and ***p < 0.001 vs. MPTP-treated vehicle control. *p* values are from a one-way ANOVA followed by Tukey’s test.

*In vivo* effects of DA-9805 were evaluated using an MPTP-injected subacute PD model mice. The experimental scheme is summarized in Fig. 5a. Rotarod tests with some modifications were performed to determine whether DA-9805 protected against the motor deficits caused by MPTP neurotoxicity. The mice injected with MPTP had a significant reduction in the latency to fall compared to normal control mice on the rotarod test (67.6 ± 30.1 sec vs. 194.6 ± 21.2 sec, *p* < 0.001) (Fig. 5b). The latent period significantly increased in groups treated with 10 mg/kg (161.4 ± 30.8 sec, *p* < 0.05) and 20 mg/kg (183.3 ± 28.2 sec, *p* < 0.01) of DA-9805 as well as in the group treated with rasagiline (150.5 ± 30.0 sec, *p* < 0.01) compared to the MPTP-treated vehicle control group. DA-9805 effectively countered MPTP-induced behavioral coordination deficiencies in a similar manner to the rasagiline-treated group, which suggests that DA-9805 may improve climbing rod behavioral deficits in mice with PD.

Dopamine levels were measured in dissected striatum to examine the effect of DA-9805 on the dopaminergic system. HPLC analysis indicated that MPTP injection significantly reduced dopamine levels to 36.8 ± 2.3% of the normal control group (0.29 ± 0.04 vs. 0.79 ± 0.12 ng/μg striatum, respectively; *p* < 0.01, n = 6 per group). Oral administration of DA-9805 (10 and 20 mg/kg) and rasagiline significantly antagonized the MPTP-induced dopamine depletion (0.49 ± 0.04, 0.51 ± 0.09, and 0.43 ± 0.03 ng/μg striatum, respectively) compared to the vehicle control (*p* < 0.05, Fig. 5c). These findings suggest that DA-9805 inhibits the MPTP-induced depletion of total striatal dopamine, which may result in improvements in motor function.

### DA-9805 protected nigrostriatal dopaminergic neurons from MPTP neurotoxicity in a subacute PD model mice

**Figure 6:**
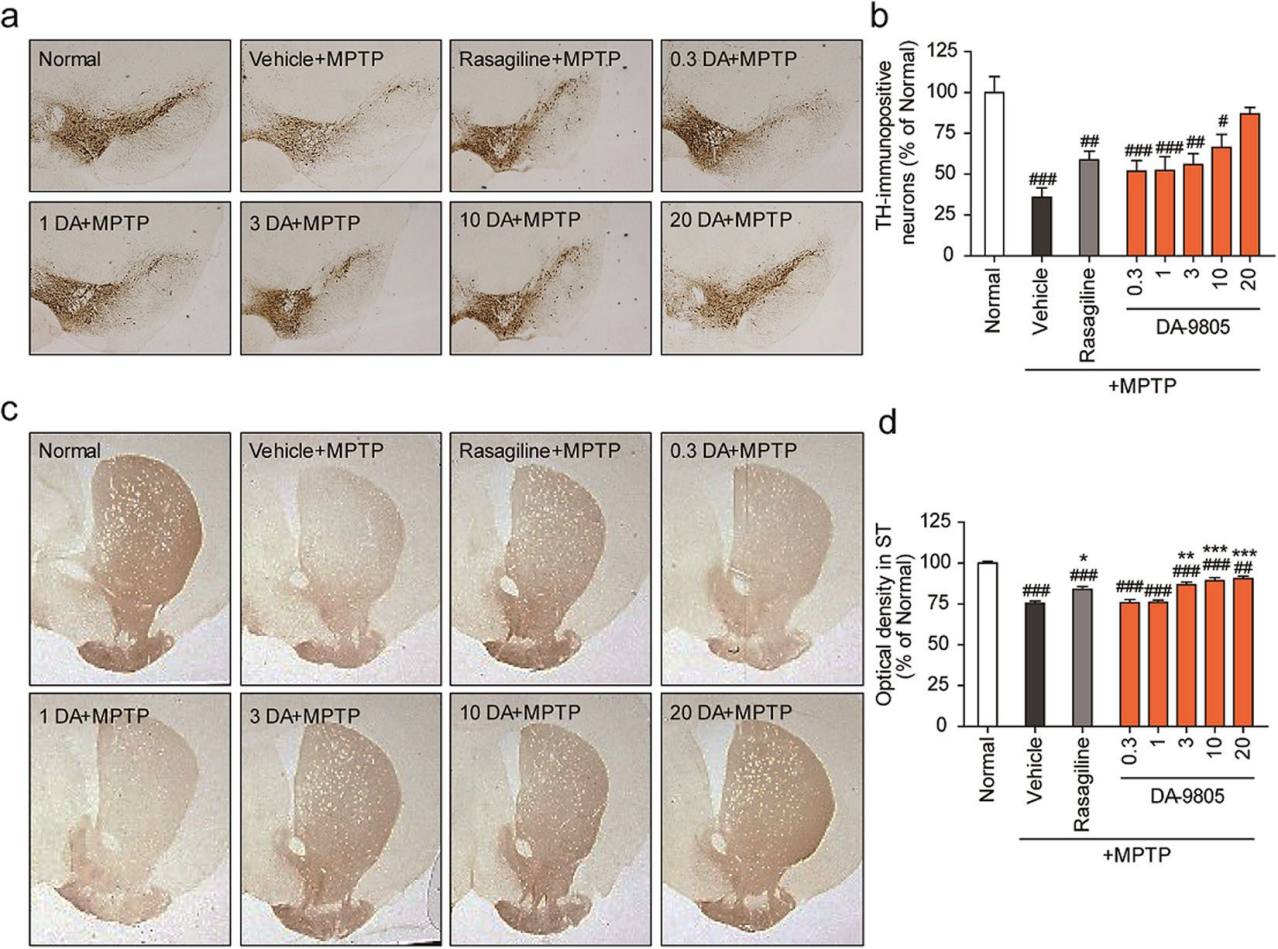
Protective effect of DA-9805 on dopaminergic neurons in an MPTP-induced subacute PD model mice. Mice were orally administered DA-9805 (0.3~20 mg/kg), rasagiline (0.05 mg/kg), or 3% HPMC vehicle for 12 days. On day 8, MPTP was injected intraperitoneally for five days (30 mg/kg, 24-h interval). Dopaminergic neurons were visualized via tyrosine hydroxylase (TH) immunohistochemistry. (**a**,**c**) Representative photomicrographs of the SNpc (**a**) and the striatum (**c**) were presented. (**b**,**d**) Quantified graphs of (**a**,**c**). (**b**) The number of TH-immunopositive neurons in the substantia nigra pars compacta (SNpc) were counted and (**d**) the optical density in the striatum was measured. The data are presented as the mean ± SEM (n = 8). ^#^*p* < 0.05, ^##^*p* < 0.01, ^###^*p* < 0.001 vs. Normal; *p < 0.05, **p < 0.01, and ***p < 0.001 vs. MPTP-treated vehicle control. *p* values are from a one-way ANOVA followed by Tukey’s test.

To investigate the effects of DA-9805 on dopaminergic neurons, mouse brains were immunostained with anti-TH antibody. Dopaminergic neuronal cells were quantified as the stereological count of TH-positive cells in the SNpc and the optical density of TH-positive fibers in the striatum. The quantitative data confirmed the depletion of TH-positive cells (dopaminergic neuronal death) in the SNpc (35.8 ± 5.8%, *p* < 0.001, n = 8) and the loss of TH-positive fibers in the striatum (75.3 ± 1.3%, *p* < 0.001) in MPTP-injected mice compared to normal control mice. Mice pre-treated with DA-9805 (10 or 20 mg/kg) exhibited a significant increase in TH-positive neurons in the SN (10 mg/kg: 66.4 ± 7.9%, *p* < 0.05; 20 mg/kg: 86.8 ± 4.0%, *p* < 0.01) and TH-positive fibers in the striatum (10 mg/kg: 89.2 ± 1.9%, *p* < 0.001; 20 mg/kg: 90.5 ± 1.4%, *p* < 0.01) compared to vehicle control mice (Fig. 6). The reference drug rasagiline also increased TH-positive cells in the SNpc (58.7 ± 5.5%, *p* < 0.05) and TH-positive fibers (83.9 ± 1.8%, *p* < 0.01), but this increase was less than that observed following DA-9805. These results collectively suggest that DA-9805 has neuroprotective effects in the MPTP-injected subacute PD model mice.

### Amelioration of both *in vitro* and *in vivo* mitochondrial damage and insulin signaling by DA-9805

**Figure 7:**
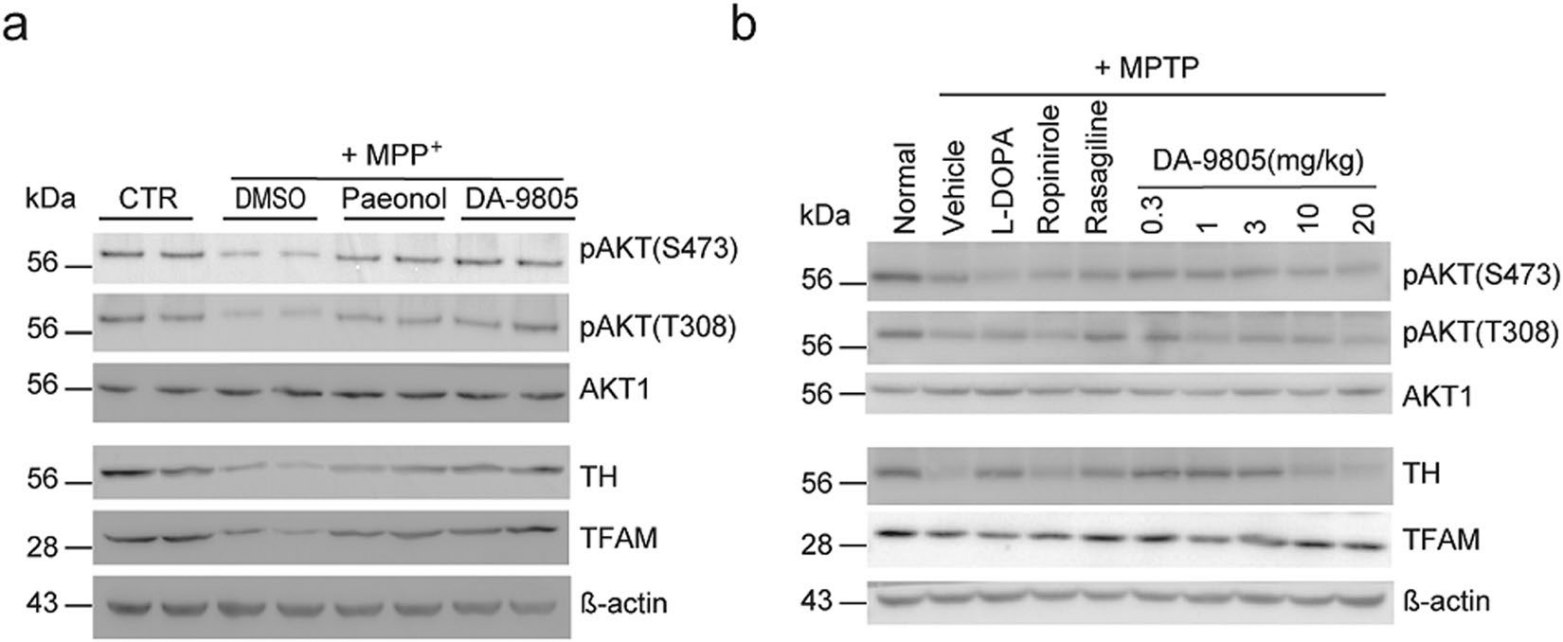
Amelioration by DA-9805 of both *in vitro* and *in vivo* mitochondrial damage and insulin signaling. (**a**) SH-SY5Y cells were pre-treated with paeonol (1 μg/ml) and DA-9805 (1 μg/ml) for 4 h, followed by incubation with 1 mM MPP^+^ for 20 h, and analyzed by Western blots using the indicated antibodies. (**b**) Mice were orally administered DA-9805 (0.3~20 mg/kg), reference drugs (50 mg/kg L-DOPA; 0.05 mg/kg ropinirole; 0.05 mg/kg rasagiline), or 3% HPMC vehicle for 12 days. On day 8, MPTP was injected intraperitoneally for five days (30 mg/kg, 24-h interval). The substantia nigra pars compacta (SNpc) was dissected from each brain and analyzed by Western blots using the indicated antibodies. The assay was repeated three times and representative blots are shown. Uncropped blots are presented in Supplementary Fig. S2.

We have previously shown that MPP^+^- or MPTP-mediated mitochondrial damage impairs AKT phosphorylation in the insulin signaling pathway and mitochondria transcription factor A (TFAM) expression in both cultured cells and SNpc^22^. Western blots to determine the levels of pAKT and AKT in SH-SY5Y cells (Fig. 7a) or SNpc (Fig. 7b) revealed that DA-9805 prevented the suppression of AKT phosphorylation at S473 and T308, and TFAM and TH expression. Paeonol also restored phosphorylation of AKT and expression of TH and TFAM. The effect of the reference compounds, ropinirole, rasagiline, and L-DOPA, on the inhibition of pAKT and TFAM were weaker than DA-9805. We conclude that DA-9805 is therapeutically effective in toxin-induced cellular and mouse models of PD by reversing or protecting against damage to mitochondria and AKT signaling.

### DA-9805 normalized the expression of mitochondrial genes in SNpc and striatum of MPTP-injected mice

**Figure 8:**
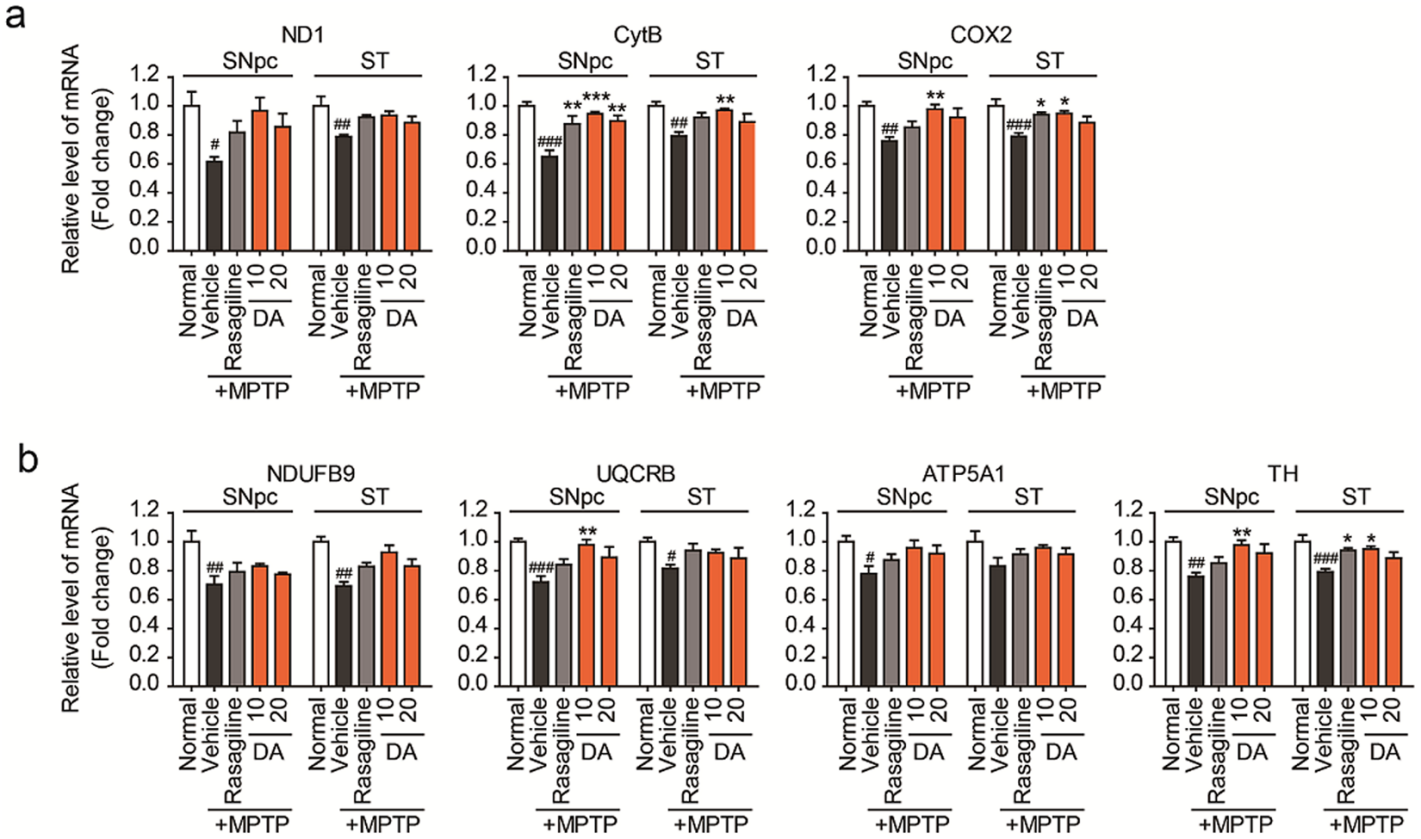
DA-9805 normalized the mRNA levels of mitochondria OXPHOS subunits in the SNpc and the striatum in an MPTP-induced subacute PD model mice. Analysis of mitochondria OXPHOS subunits via real-time quantitative PCR (qRT-PCR). Mice were orally administered DA-9805 (10 or 20 mg/kg), reference drug (0.05 mg/kg rasagiline), or 3% HPMC vehicle for 12 days. On day 8, MPTP was injected intraperitoneally for five days (30 mg/kg, 24-h interval). The substantia nigra pars compacta (SNpc) and the striatum (ST) were dissected from each brain and analyzed via qRT-PCR. (**a**) mtDNA-encoded OXPHOS subunits. (**b**) Nuclear DNA-encoded OXPHOS subunits and tyrosine hydroxylase (TH). The data are presented as the mean ± SEM (n = 7). ^#^*p* < 0.05, ^##^*p* < 0.01, ^###^*p* < 0.001 vs. Normal; *p < 0.05, **p < 0.01, and ***p < 0.001 vs. MPTP-treated vehicle control. *p* values are from a one-way ANOVA followed by Tukey’s test.

The mRNA levels of mitochondria-related genes in dissected brain tissues were determined by real-time PCR to determine the target site of DA-9805. The expressions of many mtDNA-encoded and nuclear DNA-encoded OXPHOS subunits are significantly reduced by MPP^+^ or MPTP treatment^22^. In this study, we measured complex 1 (ND1, NDUFB9), complex 3 (CytB, UQCRB), complex 4 (COX2), and complex 5 (ATP5A1). Oral administration of DA-9805 normalized the mRNA levels of both mtDNA-encoded genes (ND1, CytB, COX2) (Fig. 8a) and nuclear DNA-encoded genes (NDUFB9, UQCRB, ATP5A1) (Fig. 8b) in the SNpc and striatum of MPTP-injected mice (*p* < 0.05 vs. MPTP-vehicle control). Similar results were observed for rasagiline, but DA-9805 was more effective than rasagiline in all genes tested. Interestingly, mRNA expressions were normalized in the cerebellum but not in the cortex (data not shown).

## Discussion

The present study demonstrated that a standardized ethanol extract DA-9805, combination of MC, BR, and ADR, showed neuroprotective effects via alleviating mitochondria damages and insulin signaling disruption in experimental models of PD. Given that mitochondria are involved in the pathogenesis of neurodegenerative diseases, we propose that DA-9805 may be a suitable candidate for disease-modifying therapeutics against PD.

Neurodegenerative diseases such as PD, Alzheimer’s disease and dementia with Lewy bodies, have a multifactorial patho-etiological origin. Among the proposed underlying pathophysiological mechanisms, oxidative stress and mitochondrial dysfunction have been credited as major pathways of neurodegeneration^23,24^. Therefore, any agent that potentiates mitochondrial bioenergetics or prevents mitochondrial dysfunction should be considered a potential anti-neurodegenerative disease candidate^10^ although conventional mitochondrial medicines have not been successfully developed. Coenzyme Q10 (CoQ10) including mitochondria-targeted coenzyme Q10 (MitoQ) restored dysfunctional mitochondria and prevented the injury to nigral dopaminergic neurons^25^. Another mitochondrial energy enhancer creatine showed early promise as a possible PD medicine because of its neuroprotection from MPTP-induced animal models. In clinical trials, however, both creatine^26,27^ and CoQ10^28,29^ produced insufficient evidence to support the use for neuroprotection in PD patients. Therefore, at present, the need to identify true neuroprotective or disease-modifying agent that can slow or stop neurodegeneration has not been met^30,31^.

Traditional medicine is gaining worldwide popularity as a rich source for new drug discovery^32^. The benefits of traditional medicine could be synergized with modern evidence-based medicinal evaluation, standardization of herbal products, and randomized placebo-controlled clinical trials that support clinical efficacy. Accumulating evidence suggest that the pathogenesis of neurodegenerative diseases is very complex and there are multiple potential drug targets for PD in biochemical pathways. We therefore hypothesized that multiple targeting strategy using total extract of traditional medicine could be better than the conventional drug-discovery approach aimed at ‘one gene, one drug, one disease’ and that the constituents of DA-9805 would simultaneously target multiple dysfunctional pathways related to neurodegeneration. The current functional assay of DA-9805 provided an evaluation of neuroprotection based on modern evidence and suggested mitochondrial activity as the mechanisms of action of DA-9805.

Paeonol, an active component of MC, has been reported to protect against ER-stress-induced endothelial dysfunction via the AMPK/PPARδ signaling pathway^33^ and reduced cerebral infarction involving superoxide and microglia activation^34^. Another active component of MC, paeoniflorin, was shown to promote dopaminergic neuronal survival *in vivo* due to the inhibition of monoamine oxidase-B (MAO-B) via the PI3K/AKT signaling pathway^35^. Ethanol extract of ADR showed anti-inflammatory effect by inhibiting cycloxygenase, and NF-κB pathway^36,37^ and anti-oxidative effects screened by the 2,2-diphenyl-1-picryl-hydrazyl-hydrate (DPPH) assay^38^. Imperatorin is a major active ingredient of ADR which shows anti-inflammatory effects and neuroprotective effect against ischemia-reperfusion injury^39^. In addition, saikosaponins and ethanol extract of BR have been reported to reduce neuroinflammation-mediated neurodegeneration by suppressing NF-κB-mediated inflammatory pathways^18^. It is fortunate that paeonol, paeoniflorin, imperatorin, and saikosaponins are the most abundant active compounds in DA-9805 because they are useful as markers for quality control and pharmacological efficacy. In fact, the chemical fingerprint of DA-9805 presented in Fig. 1 is useful for manufacturing control to ensure batch-to-batch consistency. Furthermore, simultaneous determination of paeonol, imperatorin and saikosaponin A was successfully performed in rat plasma after oral administration of DA-9805, which is useful for pharmacokinetic study of DA-9805^40^.

Toxins like MPTP are known to cause symptoms of parkinsonism in animals and humans. It has been repeatedly demonstrated that MPTP is the gold standard for toxin-based PD animal models because it recapitulates the primary pathological and biochemical features of PD, such as oxidative stress, mitochondrial dysfunction, and apoptosis^41,42^. Subacute treatment of MPTP induces significant loss of dopaminergic neurons in the nigrostriatal pathway, which play important roles in motor function. We observed that DA-9805 produced an effective rescue of dopaminergic neuronal death (Figs 2 and 6) and related behavioral deficits (Fig. 5). DA-9805 also inhibited striatal dopamine depletion in MPTP-injected mice (Fig. 5c). Recovery of dopamine levels may be due to direct dopamine supplementation from rescued dopaminergic neurons. Our other findings revealed, however, that DA-9805 also rescued toxin-induced mitochondrial damage in both *in vitro* and *in vivo* models (Fig. 7). Indeed, DA-9805 reduced several aspects of MPP^+^-induced mitochondrial dysfunction, including decreased ATP contents, dissipation of mitochondrial membrane potential, increased production of mitochondrial and intracellular ROS (Fig. 3, Supplementary Fig. S1), and reduced OCR (Fig. 4). Previously, we have shown that mitochondrial activation by TFAM or constitutively active myr-AKT abrogated MPP^+^-mediated damages on mitochondria and insulin signaling, leading to recovery of nigrostriatal neurodegeneration^22^. Since AKT and insulin signaling pathways are dysregulated in the brain of PD patients^43,44^, insulin-sensitizing agents have been proposed as potential candidates for neuroprotection^45,46^. DA-9805 significantly enhanced TFAM expression, AKT phosphorylation (Fig. 7) and OXPHOS subunit expression (Fig. 8), followed by mitochondrial activation and neuroprotection (Fig. 2). When we compared efficacy of DA-9805 with reference drugs such as creatine, rasagiline (a selective inhibitor of MAO-B), and ropinirole (a dopamine agonist), DA-9805 exhibited superior protection against MPP^+^-induced mitochondrial dysfunction (Figs 3 and 4). It suggests that the neuroprotective effect of DA-9805 may act primarily through mitochondrial activation instead of directly supplementing dopamine. In future studies, it is necessary to validate efficacy using another PD animal model such as the alpha-synuclein pre-formed fibril animal model and study the detailed mode of action at the molecular level.

In conclusion, the present study demonstrated that DA-9805 ameliorated MPTP-induced behavioral abnormalities and dopaminergic neuronal loss in C57BL/6 mice. It was revealed that this effect of DA-9805 was closely associated with the improvement of mitochondria function. DA-9805 may serve as a promising complementary and/or preventive therapy to slow or halt the progression of PD.

## Methods

### Materials

MC, BR and ADR were commercially purchased from Kyungdong Herbal Market (Seoul, Korea) (see Supplementary Material for details of each herb). The primary antibodies against tyrosine hydroxylase (TH), AKT, pAKT(T308), and pAKT(S473) were purchased from Cell Signaling Technology (Beverly, MA, USA). Anti-β-actin antibody was purchased from Sigma-Aldrich Co. (St. Louis, CO, USA). Horseradish peroxidase (HRP)-conjugated secondary antibodies were purchased from Cell Signaling Technology. Biotinylated anti-rabbit antibody and the avidin-biotin peroxidase complex (ABC) standard kit were purchased from Vector Laboratories (Burlingame, CA, USA). Calcein AM, tetramethylrhodamineethylester (TMRE), 5,6-chloromethyl-2′,7′-dichlorodihydrofluorescein diacetate acetyl ester (CM-H_2_DCFDA), and MitoSox agents were purchased from Molecular Probes (Eugene, OR, USA). SH-SY5Y human neuroblastoma cells were purchased from ATCC® (CRL-2266™; Manassas, VA, USA). All other reagents were purchased from Sigma-Aldrich. Media and culture reagents were products of Gibco Industries Inc. (Auckland, New Zealand).

### Preparation of DA-9805

The dried and meshed plant material mixture of MC, BR, and ADR at a 1:1:1 ratio (weight) were extracted with 90% ethanol on a stirring plate for 24 h at room temperature (15 to 25 °C) and filtered. Ethanol filtrate was evaporated under reduced pressure at 45 to 50 °C. The viscous ethanol extract of the mixture was freeze-dried to obtain powdered extract and kept at 4 °C (yield 6.7%) until use.

### Quality standardization of DA-9805

DA-9805 was standardized based on the contents of paeonol, saikosaponin A, and imperatorin. DA-9805 (10 mg) was dissolved in 10 mL of 90% ethanol and centrifuged at 13,000 rpm for 5 min. The contents of the active components in the supernatant were analyzed using a Dionex UltiMate 3000 high-performance liquid chromatography (HPLC) system (Thermo Fisher Scientific Inc., Waltham, MA, USA) equipped with an UltiMate 3000 pump and UltiMate 3000 photodiode array detector. The standard solutions were prepared using appropriate amounts of paeonol (6.0 mg), imperatorin (7.0 mg) and saikosaponin A (6.0 mg) dissolved in 100 mL ethanol.

An INNO C18 column (4.6 × 250 mm, 5 μm, Young Jin Biochrom Co., Ltd. Seoul, Korea) was used to detect paeonol using the following chromatographic conditions. The mobile phase was comprised of phosphoric acid (solvent A, pH 3.0) and acetonitrile (MeCN, solvent B) at a flow rate of 1.0 mL/min, an injection volume of 10 μL, detection at UV 274 nm, and a column oven temperature of 30 °C. The HPLC operating conditions were programmed to yield the following: at 0 min, 10% B; at 0–13 min, a linear increase to 30% B; at 13–36 min, a linear increase to 35% B; at 36–41 min, a linear increase to 90% B; at 41–50 min, 90% B; at 50–51 min, a linear decrease to 10% B; at 51–60 min, 10% B. The same INNO C18 column was used for detection of imperatorin using the following chromatographic conditions. The mobile phase was comprised of double distilled water (solvent A) and methanol (solvent B) in a linear gradient mode at a flow rate of 1.0 mL/min, an injection volume of 10 μL, detection at UV 254 nm, and a column oven temperature of 30 °C. The HPLC operating conditions were programmed to yield the following: at 0–20 min, 56% B; at 20–50 min, a linear increase to 70% B; at 50–51 min, a linear increase to 90% B; at 51–60 min, 90% B; at 60–61 min, a linear decrease to 56% B; at 61–70 min, 56% B. A ZORBOX Eclipse XDB-C18 column (4.6 × 250 mm, 5 μm, Agilent, Santa Clara, CA, USA) was used for detection of saikosaponin A using the following chromatographic conditions. The mobile phase was comprised of solvent A, phosphoric acid, with a pH of 3.0 and solvent B, MeCN, at a flow rate of 1.0 mL/min, an injection volume of 20 μL, detection at UV 203 nm, and a column oven temperature of 30 °C. The HPLC operating conditions were programmed to yield the following: at 0–35 min, 33% B; at 35–36 min, a linear increase to 90% B; at 36–45 min, 90% B; at 45–46 min, a linear decrease to 33% B; at 46–50 min, 33% B.

### UHPLC fingerprinting of DA-9805

To obtain stable and reproducible chromatographic fingerprints of DA-9805 for quality control, UHPLC fingerprint validation analysis was performed on the basis of the retention times and peak areas of eight marker ingredients: gallic acid, chlorogenic acid, paeoniflorin, paeonol, oxypeucedanin, saikosaponin A, imperatorin, and isoimperatorin. Among three batches of DA-9805 (MB1601, MB1602, and MB1603), MB1601 was assigned as a standard. Validation was performed according to recent guidelines for specificity, linearity, precision, accuracy, and robustness (ICH Harmonized Tripartite Guideline, adapted in 1994 and amended in 2006).

The analysis was performed on a Thermo Ultimate 3000 ultra-HPLC (UHPLC) system with a XBridge C18 column (4.6 × 250 mm, 100 Å, 5 μm, Waters, Milford, MA, USA) at 30 °C using a gradient solvent system at a rate of 1.5 mL/min comprised of 0.01 M phosphoric acid (solvent A) and MeCN (solvent B). The gradient profile was as follows: at 0 min, 10% B; at 0–10 min, a linear increase to 20% B; at 10–10.1 min, a linear increase to 25% B; at 10.1–30 min, a linear increase to 33% B; at 30–48 min, a linear increase to 70% B; at 48–48.1 min, a linear increase to 90% B; at 48.1–54 min, 90% B; at 54–54.1 min, a linear decrease to 10% B; at 54.1–60 min, 10% B. Various Wavelength Detection (VWD) was performed as follows: at 0–5 min, 203 nm; at 5–9 min, 280 nm; at 9–18 min, 254 nm; at 18–25 min, 330 nm; at 25–30 min, 254 nm; at 30–60 min, 203 nm. The column injection volume was 5 μL. DA-9805 (10 mg/mL) was prepared in 70% MeCN. A standard solution was prepared from eight ingredients dissolved in 100 mL 70% acetonitrile to generate the following final concentrations: gallic acid (0.7 mg/ml), chlorogenic acid (0.7 mg/ml), paeoniflorin (3 mg/ml), paeonol (5 mg/ml), oxypeucedanin (0.7 mg/ml), saikosaponin A (0.3 mg/ml), imperatorin (0.6 mg/ml), and isoimperatorin (0.6 mg/ml).

### Cell cultures

SH-SY5Y cells were cultured in Dulbecco’s Modified Eagle Medium (DMEM)/F12 supplemented with 10% fetal bovine serum (FBS), 100 U/mL penicillin, and 100 μg/mL streptomycin at 37 °C with 5% CO_2_. Cells seeded at 5 × 10^4^ cells/well were cultured in 96-well plates for 24 h followed by incubation in serum-deficient media (SDM, DMEM/F12 containing 0.5% FBS) for 16 h. Cells in SDM were pre-treated with DA-9805 (0.0001~10 μg/mL) for 4 h, followed by incubation with 1 mM MPP^+^ or dimethyl sulfoxide (DMSO) vehicle for 20 h. Creatine (50 μM), rasagiline (100 nM), and ropinirole (50 μM) were treated as reference controls.

### Cell-based mitochondrial activity and apoptosis assays

Cell-based mitochondrial activity assays were performed using SH-SY5Y cells in 96-well black plates with clear bottom as described previously in detail^22,47^. The assays cover the quantitative assays for cell viability (calcein), complex 1, methyl thiazyl tetrazolium-mitochondrial dehydrogenase activity (MTT), mitochondrial membrane potential (TMRE, Molecular Probe, Eugene, OR, USA), ATP contents, and CM-H_2_DCFDA- or MitoSox (Molecular Probe)-dependent reactive oxygen species (ROS). The fluorescence intensities at 550 nm/580 nm for TMRE, at 510 nm/580 nm for Mito Sox, or 494 nm/522 nm for DCF-DA were normalized by Hoechst intensity at 355 nm/480 nm (Spectramax Gemini EM, Molecular Devices, Sunnyvale, CA, USA). The Caspase-Glo3/7 assay system (Promega Co, Madison, WI, USA) was used for caspase detection according to the manufacturer’s instructions. Briefly, the drug-pretreated SH-SY5Y cells were exposed to 1 mM MPP^+^ for 24 h, and subsequently incubated with the Caspase-Glo reagent containing proluminescent DEVD tetrapeptide substrate for 1 h. The luminescent intensity was measured by a luminometer (Lumat LB 9501/16, Berthold, Wildbad, Germany).

### Measurement of endogenous cellular oxygen consumption rate (OCR)

OCR was measured in adherent cells using a Seahorse XF-24 Analyzer (Seahorse Bioscience, Billerica, MA, USA) following the manufacturer’s protocol with minor modifications^48^. SH-SY5Y cells (5.0 × 10^3^ cells/well) in XF-24 microplates incubated in 250 μL DMEM containing 10% FBS at 37 °C at an atmosphere of 5% CO_2_ for 24 h. Assays were performed by replacing DMEM without sodium bicarbonate pre-warmed to 37 °C, the OCR (basal OCR) was measured simultaneously for 3 min. Oligomycin, carbonyl cyanide-p-trifluoromethoxyphenylhydrazone (FCCP), and rotenone were consecutively injected into each well to reach the desired final working concentrations, 1 μg/mL, 0.3 μM, and 0.1 μM, respectively. The OCR was calculated from 3-min measurement cycles and normalized to the cell number. The ATP turnover rate (basal OCR – oligomycin-OCR) and total respiratory capacity (FCCP-OCR – rotenone-OCR) were calculated.

### Western blot analysis

Cell lysates or tissue strip lysates were prepared on ice in PRO-PREP lysis buffer (10 mM HEPES, pH 7.9, 10 mM KCl, 2 mM MgCl_2_, 0.5 mM dithiothreitol, 1 mM phenylmethylsulfonyl fluoride, 1 μg/mL aprotinin, 1 μg/mL pepstatin A, and 2 μg/mL leupeptin; iNtRON Biotechnology, Gyeonggi-do, Korea). Protein extracts (20~30 μg) were separated on 12–15% SDS-PAGE and analyzed via Western blot followed by an enhanced chemiluminescence system (ECL, Amersham Bioscience, Piscataway, NJ, USA). The following working dilutions were used: anti-TH (1:1000), anti-AKT (1:1000), and anti-pAKT (1:1000). HRP-conjugated secondary antibodies were diluted to 1:3000 for anti-mouse IgG and 1:2000 for anti-rabbit IgG. Rabbit polyclonal antibodies against human TFAM (1:2000) were prepared in our laboratory and used for TFAM detection^49^. Anti-β-actin antibody (1:3000) was used as a loading control.

### Animals

Male C57BL/6 mice aged 8–10 weeks with initial body weight of 20–24 g were purchased from Samtako Bio Korea Co. Ltd (Osan-si, Korea) and allowed at least 1 week of acclimatization before being subjected to the study. The animals were housed in a regulated environment with a 12-h/12-h light/dark cycle. Food and water were provided *ad libitum*. All the animal experiments were carried out in accordance with the National Institutes of Health Guide for the Care and Use of Laboratory Animals (NIH Publications No. 80–23; revised 1996). The protocols were approved by Institutional Animal Care and Use Committee of Dong-A ST (I1408025).

### MPTP-injected subacute PD mice and drug administration

A 3% hydroxypropylmethylcellulose (HPMC, Shin-Etsu, Japan) solution was prepared by dissolving 3 g of HPMC in 100 mL of distilled water under magnetic stirring for 12 h. DA-9805 and rasagiline was dissolved in 3% HPMC solution. MPTP was dissolved in distilled water. Mice (n = 56 per experiment) were randomly divided into the following groups (n = 8 per group): (1) Normal control (intraperitoneally injected distilled water plus intraorally administered 3% HPMC solution), (2) MPTP-vehicle control (MPTP plus 3% HPMC solution), (3) MPTP + rasagiline (0.05 mg/kg), (4) MPTP + DA-9805 (0.3 mg/kg), (5) MPTP + DA-9805 (1 mg/kg), (6) MPTP + DA-9805 (3 mg/kg), (7) MPTP + DA-9805 (10 mg/kg), and (8) MPTP + DA-9805 (20 mg/kg). DA-9805 (0.1 mL/10 g body weight) or rasagiline (0.05 mg/kg/day) was intragastrically administered to mice at designated doses for 12 days. In some cases, L-DOPA (50 mg/kg) and ropinirole (0.05 mg/kg) were administered instead of DA-9805 as reference control drugs. All groups except for the normal control group were administered MPTP (30 mg/kg/day) intraperitoneally for five days from day 8 of the 18-day experiment period. For Western blots, RNA, and dopamine quantifications, the indicated brain regions were isolated, immediately frozen under liquid nitrogen, and kept at −80 °C until use.

### Rotarod test

To determine forelimb and hindlimb motor coordination and balance, we performed the rotarod test as described previously^21,50^. Animals were pre-trained on the rotating bar of a rotarod unit set (LE 8500, Letica, Spain) on days 15 and 16 before the test on day 17. During pre-training, three trials per day were performed (5 rpm rotation speed on the first day, 15 rpm rotation speed on the second day). Mice were kept on the rotating bar (7.3-cm diameter) for 5 min on each trial. On day 17, the time spent on the rotating bar at 20 rpm, which was defined as the latent period, was recorded. Performance was recorded as 300 s if the latent period exceeded 300 sec. Mice had at least 5 min of rest between trials to reduce stress and fatigue. Each animal underwent three test trials, and the mean of the test results was subjected to statistical analysis.

### Determination of striatal dopamine content

The dopamine content in the striatal samples was analyzed with HPLC (Dionex Corp., Sunnyvale, CA, USA) equipped with an electrochemical detecting system (ESA Coulochem III detection system, range 100 nA, potential −50 mV to +300 mV; ESA Inc., Chelmsford, MA, USA). The striatum was identified (bregma 0.98–0.50 mm) according to the mouse brain atlas^51^ and dissected using previously reported methods^20,21^. Briefly, samples were homogenized on ice in 40 μL of 0.2 M perchloric acid and then centrifuged for 20 min at 4 °C at 12,000 rpm. The supernatant was filtered through a 0.22-μm membrane, and an aliquot (10 μL in volume) of the resulting solution was injected into the HPLC pump. Chromatographic separation was performed using a C18 reverse-phase column at 25 °C, and data were analyzed using Chromeleon^TM^ software (Version 6.40) from Dionex Corp. Dopamine standards were prepared in 0.2 M perchloric acid, and each concentration was quantified from a standard curve. The levels were calculated as nanograms per microgram of total protein, which was determined by the Bradford’s protein assay (Bio-Rad, Hercules, CA, USA).

### Immunohistochemistry of TH-positive neurons

On day 18, mice were anesthetized with sodium pentobarbital (50 mg/kg intraperitoneally) and transcardially perfused with a saline solution containing 0.5% sodium nitrate and heparin (10 U/mL) and then fixed with 4% paraformaldehyde dissolved in 0.1 M phosphate-buffered saline (PBS). Brains were dissected from the skull, post-fixed overnight in buffered 4% paraformaldehyde at 4 °C, and stored in a 30% sucrose solution until they sank. Subsequently, the brains were prepared for frozen section on Cryostat (Microsystems AG, Leica, Wetzlar, Germany) in 30-µm thick coronal sections, and rinsed in PBS. The tissue sections were incubated overnight at 4 °C with primary rabbit anti-TH antibodies followed by incubation in ABC solution for 1 h at room temperature. The peroxidase activity was visualized by incubating with 0.05% DAB and 0.003% hydrogen peroxide in 0.1 M PBS. After the labeled tissue sections were mounted on gelatin-coated slides, the TH-immunopositive cells in the SNpc on the coverslip were counted at x100 magnification under a bright-field microscope (Olympus Optical, Carl Zeiss, Peabody, MA, USA) under blinded condition by two independent observers. TH-immunopositivity in the striatum was measured at x40 magnification using ImageJ software (National Institutes of Health, Bethesda, MD, USA). Data are presented as the percentages of the control group values.

### RNA preparation and real-time qPCR

The total RNA of the dissected brain tissue was isolated with Trizol reagent (Invitrogen, Carlsbad, CA, USA). Total RNA (1.5 μg) was reverse transcribed using MMLV reverse transcriptase (Promega, Madison, WI, USA), RNasin Ribonuclease inhibitors (Promega), 10 ng of random primers (Invitrogen), and 25 mM of dNTP mix (Gene Craft, Ludinghausen, Germany), according to the manufacturer’s instructions. Real-time quantitative RT-PCR (qRT-PCR) was performed using primers for ND1 (5′-tac gag ccg tag ccc aaa ca-3′ and 5′-gat cgt aac gga agc gtg ga-3′ for murine), CytB (5′-tga ggg ggc ttc tca gta ga-3′ and 5′-tga gcg tag aat ggc gta tg-3′ for murine), COX2 (5′-ttg gtc tac aag acg cca ca-3′ and 5′ cgg tta ata cgg ggt tgt tg–3′ for murine), NDUFB9 (5′-caa ggt tcc aga atg gtg ct-3′ and 5′-agg agg caa agc ttc agt ca-3′ for murine), UQCRB (5′-agg ttt ttc gcg aga ctg ag-3′ and 5′-ttg ccc cat gtg tag atg ag-3′ for murine), ATP5A1 (5′-aca gtc aga cgc aaa gct ca-3′ and 5′-acc acc aca caa atc ctg gt-3′ for murine), TH (5′-cag ctg gag gat gtg tct ca-3′ and 5′-ata ggt gag gca tga cg-3′ for murine) and 18 S rRNA (5′-gag cga aag cat ttg cca ag-3′ and 5′-ggc atc gtt tat ggt cgg aa-3′ for both human and murine) on a Roter-Gene Q (Qiagen, Hilden, Germany) with 2x AmpiGene^®^ qPCR Green Mix Lo-ROX (Enzo Biochem, NY) at 95 °C for 10 min, followed by 45 cycles of 95 °C for 5 s and 60 °C for 15 s and 72 °C for 20 sec. Measurements were performed in duplicate for each sample. Quantity of mRNA was corrected by simultaneous measurement of nuclear DNA encoding 18 S rRNA. The relative quantification in gene expression was determined using the 2−^ΔΔCt^ method. The relative mRNA expression levels were presented as fold changes compared to the control condition.

### Statistical Analyses

The results are presented as the mean ± standard error of the mean (SEM). Statistical significances between experimental groups were evaluated by one-way ANOVA with Tukey post-hoc testing analysis using InStat (GraphPad Software, San Diego, CA, USA). Values of *p* < 0.05 were considered statistically significant.

## Electronic supplementary material

Supplementary Information
