# Supplementary material for: Triple herbal extract DA-9805 exerts a neuroprotective effect via amelioration of mitochondrial damage in experimental models of Parkinson’s disease
Source: Sci Rep. 2018 Oct 29;8:15953. doi: 10.1038/s41598-018-34240-x (PMC6206089; doi:10.1038/s41598-018-34240-x)
Supplement: Supplementary file 1 — Supplementary Information [file 41598_2018_34240_MOESM1_ESM.pdf]

Supplementary Information

Triple herbal extract DA-9805 exerts a neuroprotective effect via amelioration of mitochondrial damage in experimental models of Parkinson’s disease

Jin Seok Jeong[1,2], Ying Piao[3#], Sora Kang[3], Minuk Son[3], Young Cheol Kang[3], Xiao Fei Du[1], Jayoung Ryu[1], Young Woong Cho[1], Hai-Hua Jiang[1], Myung Sook Oh[4], Seon-Pyo Hong[4], Young J. Oh[2,*], Youngmi Kim Pak[3,5*]

[1]R&D Center of Dong-A ST, Yong-in, Kyungki-do 17073, Korea

[2]Department of Systems Biology, College of Life Science and Biotechnology, Yonsei University, Seoul 03722, Korea

[3]Department of Neuroscience, Graduate School, [4]Department of Oriental Pharmaceutical Science, College of Pharmacy, [5]Department of Physiology, College of Medicine, Kyung Hee University, Seoul 02447, Korea

Supplementary Figures

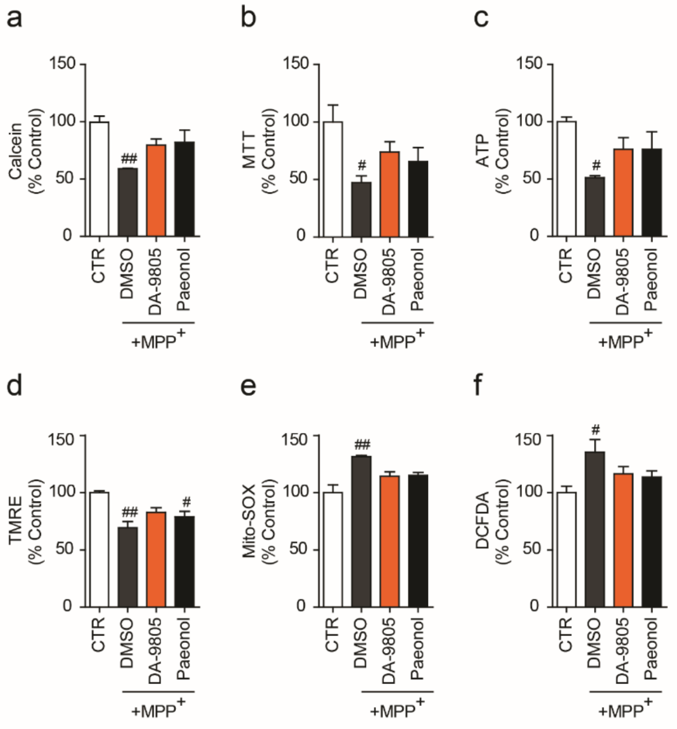

Supplementary Fig. S1. DA-9805 protects MPP$^+$-damaged mitochondrial activity in primary cortical neuronal cells. Primary cortical neuronal cells (Piao et al., 2012) were pre-treated with paeonol (PA, 1 μg/ml) and DA-9805 (1 μg/ml) for 4 h, followed by incubation with 0.1 mM MPP$^+$ for 20 h. (a) Calcein assay. (b) MTT assay (Complex 1 activity). (c) ATP assay. (d) TMRE, mitochondrial membrane potential. (e) MitoSox for mitochondrial reactive oxygen species (ROS). (F) DCF-DA for total ROS. All values are reported as a percentage of the control (CTR). The data are plotted as the mean ± standard error of the mean (SEM) (n=3). Different letters indicate a statistically significant difference from each other.

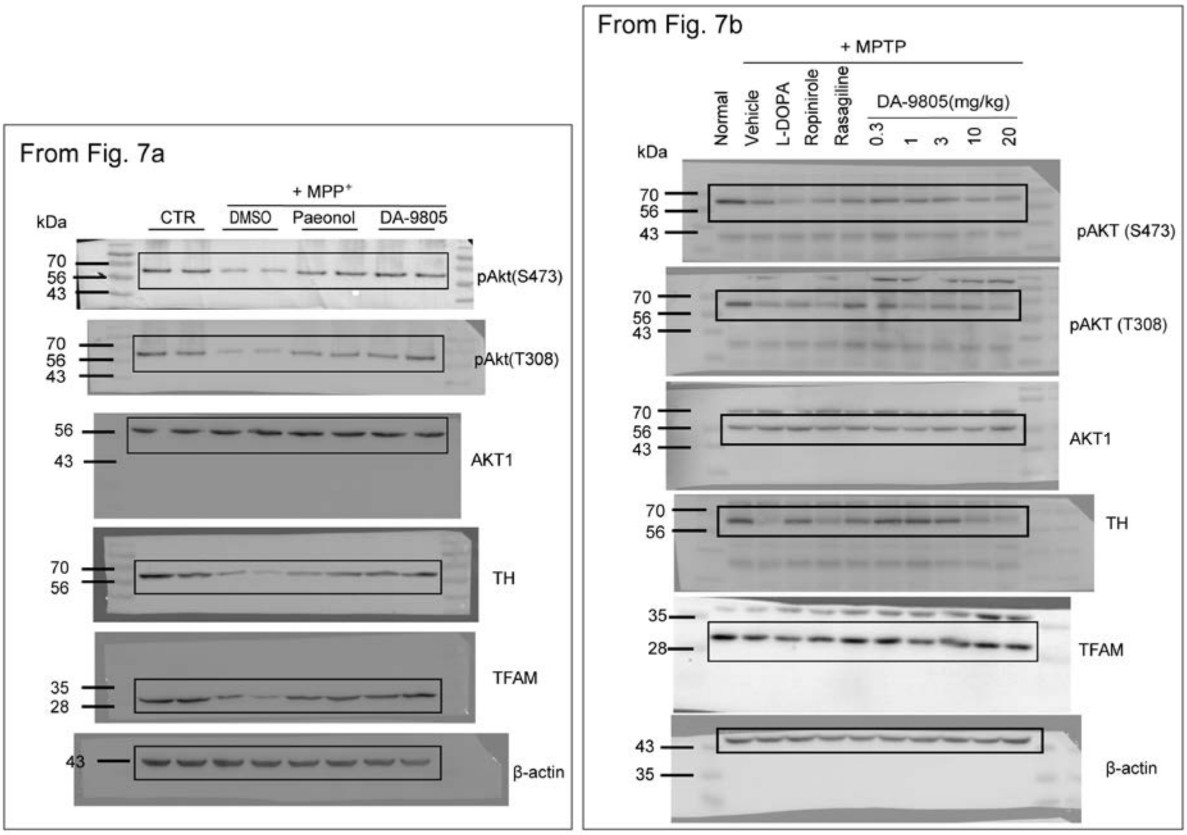

Supplementary Fig. S2. Full uncropped images. Boxes on Western blots represent the uncropped images used in Fig. 7.

Supplementary materials

MC, BR and ADR were commercially purchased from Kyungdong Herbal Market (Seoul, Korea). The growing districts and harvest times of each plant are summarized as following:

Plant	Producer	Growing district	Harvest time
Moutan cortex (MC)	Anhui Xiehecheng Pharmaceuticals, China	Shibali standardized agriculture demonstration zone, QiaoCheng District, Hao Zhou city, An Hui Province, China	Aug.-Sep.
Angelica Dahurica root (ADR)	Anhui Xiehecheng Pharmaceuticals, China	Shibali standardized agriculture demonstration zone, QiaoCheng District, Hao Zhou city, An Hui Province, China	Aug.-Sep.
Bupleurum root (BR)	Anhui Xiehecheng Pharmaceuticals, China	QiaoCheng District, Hao Zhou city, An Hui Province, China	Sep.-Oct.
